# β-Arrestin1 and β-Arrestin2 Are Required to Support the Activity of the CXCL12/HMGB1 Heterocomplex on CXCR4

**DOI:** 10.3389/fimmu.2020.550824

**Published:** 2020-09-18

**Authors:** Gianluca D’Agostino, Marc Artinger, Massimo Locati, Laurent Perez, Daniel F. Legler, Marco E. Bianchi, Curzio Rüegg, Marcus Thelen, Adriano Marchese, Marco B. L. Rocchi, Valentina Cecchinato, Mariagrazia Uguccioni

**Affiliations:** ^1^Faculty of Biomedical Sciences, Institute for Research in Biomedicine, Università della Svizzera italiana, Bellinzona, Switzerland; ^2^Biotechnology Institute Thurgau (BITg) at the University of Konstanz, Kreuzlingen, Switzerland; ^3^Humanitas Clinical and Research Center IRCCS, Rozzano, Italy; ^4^Department of Medical Biotechnologies and Translational Medicine, University of Milan, Milan, Italy; ^5^Theodor Kocher Institute, University of Bern, Bern, Switzerland; ^6^Division of Genetics and Cell Biology, Vita-Salute San Raffaele University, Milan, Italy; ^7^Department of Oncology, Microbiology and Immunology, Faculty of Science and Medicine, University of Fribourg, Fribourg, Switzerland; ^8^Department of Biochemistry, Medical College of Wisconsin, Milwaukee, WI, United States; ^9^Department of Biomolecular Sciences, Biostatistics Unit, University of Urbino, Urbino, Italy; ^10^Department of Biomedical Sciences, Humanitas University, Milan, Italy

**Keywords:** cell migration, CXCR4, CXCL12, HMGB1, β-arrestin, CXCL12/HMGB1 heterocomplex

## Abstract

The chemokine receptor CXCR4 plays a fundamental role in homeostasis and pathology by orchestrating recruitment and positioning of immune cells, under the guidance of a CXCL12 gradient. The ability of chemokines to form heterocomplexes, enhancing their function, represents an additional level of regulation on their cognate receptors. In particular, the multi-faceted activity of the heterocomplex formed between CXCL12 and the alarmin HMGB1 is emerging as an unexpected player able to modulate a variety of cell responses, spanning from tissue regeneration to chronic inflammation. Nowadays, little is known on the selective signaling pathways activated when CXCR4 is triggered by the CXCL12/HMGB1 heterocomplex. In the present work, we demonstrate that this heterocomplex acts as a CXCR4 balanced agonist, activating both G protein and β-arrestins-mediated signaling pathways to sustain chemotaxis. We generated β-arrestins knock out HeLa cells by CRISPR/Cas9 technology and show that the CXCL12/HMGB1 heterocomplex-mediated actin polymerization is primarily β-arrestin1 dependent, while chemotaxis requires both β-arrestin1 and β-arrestin2. Triggering of CXCR4 with the CXCL12/HMGB1 heterocomplex leads to an unexpected receptor retention on the cell surface, which depends on β-arrestin2. In conclusion, the CXCL12/HMGB1 heterocomplex engages the β-arrestin proteins differently from CXCL12, promoting a prompt availability of CXCR4 on the cell surface, and enhancing directional cell migration. These data unveil the signaling induced by the CXCL12/HMGB1 heterocomplex in view of identifying biased CXCR4 antagonists or agonists targeting the variety of functions it exerts.

## Introduction

Chemokines are a family of chemoattractant cytokines that orchestrate the recruitment and the positioning of immune cells via G protein-coupled receptors (GPCRs), under homeostatic, or pathological conditions. It is now well established that some chemokines can form heterocomplexes with other chemokines thereby contributing to modulate chemokine activities by enhancing the effect of a low agonist dose on a selective GPCR (1–5). In addition, the chemokine CXCL12 can also form a heterocomplex with the alarmin HMGB1 (6, 7), which enhances the recruitment of human and mouse cells to inflammatory and regenerative sites via the chemokine receptor CXCR4 (6, 8–10). However, while the relevance of the CXCL12/HMGB1 heterocomplex has been demonstrated *in vitro* and *in vivo*, little is known on the molecular mechanisms elicited by its activity on CXCR4 compared to CXCL12 alone.

Chemokine receptor signaling controlling cell migration is mostly dependent on heterotrimeric G_*i*_ protein activation, while receptor desensitization and endocytosis is linked to the activity of β-arrestins. This process has been demonstrated to be far more complex since β-arrestins can directly mediate chemokine receptor signaling, sustaining important cellular responses, including cytoskeleton remodeling, and chemotaxis (11, 12). Depending on the tissue context, cell type, and chemokine receptor engaged, a given agonist could specifically activate one or both pathways, defining a “biased” or “balanced” signaling (13–15).

Almost 35% of current FDA approved therapeutics target the superfamily of GPCRs, which are involved in a variety of essential processes in physiological and pathological conditions (16). Different approaches to block the activity of GPCRs have been developed, including biased or unbiased antagonist drugs. So far, AMD3100 is the only CXCR4 unbiased antagonist approved for the use in the clinic, which blocks the activity of both G proteins and β-arrestins, and is also able to inhibit the function of the CXCL12/HMGB1 heterocomplex (6). To avoid development of tolerance induced by the use of AMD3100, other approaches to block the activity of CXCR4 have been exploited including the use of peptides with biased activity (17). In addition, the emerging concept that β-arrestins can support both G-protein-dependent and -independent signaling pathways (18) has fostered our research for a better understanding of the role of β-arrestins in promoting the activity of the CXCL12/HMGB1 heterocomplex.

The activity of CXCL12, the agonist of CXCR4, has been widely studied and the chemokine is shown to be essential in physiology, development, inflammation, and in cancer metastatic spreading to bone, lungs and brain (19). CXCR4 signaling induced by CXCL12 occurs mainly in a G protein dependent manner, promoting calcium release and activation of different kinases, including MAPKs and PI3K/Akt. These are essential cellular events which sustain cell survival, proliferation (20), and directional cell migration, the latter involving cytoskeleton remodeling and actin polymerization (21). In addition, CXCL12 is able to signal through β-arrestins in a G protein independent manner, thus defining this chemokine as a “balanced” agonist. Indeed, it has been described that CXCL12 induced chemotaxis of T cells requires a β-arrestin2-dependent activation of p38, a member of the MAPKs (22, 23). The oligomeric state of CXCL12 strongly affects its signaling and β-arrestins recruitment profiles, with only the monomeric form inducing chemotaxis, cytoskeleton rearrangements, and β-arrestin2 mobilization compared to the dimeric CXCL12 (24, 25). Moreover, β-arrestin1, by complexing with STAM-1, a member of the ESCRT-0 machinery important for CXCR4 regulation, sustains focal adhesion kinases (FAK) activation and cell migration (26).

The activity of chemokine receptors is regulated through processes of internalization and intracellular trafficking, which leads either to degradation or recycling to the plasma membrane depending on the receptor and ligand involved (12, 27, 28). In particular, after CXCL12 triggering, CXCR4 is rapidly phosphorylated by GPCR kinases (GRKs) at the C-terminus (29), ubiquitinated by AIP4 (30, 31), and internalized in a clathrin-dependent and β-arrestin dependent manner, in order to promote receptor degradation and switch off the response (32). Once internalized, CXCR4 undergoes endosomal sorting through the ESCRT-0 machinery, requiring β-arrestin1/STAM-1 interaction for HRS ubiquitination, an essential event for the regulation of CXCR4 sorting into the degradative pathway (33).

HMGB1 is a nuclear protein that can also act as a redox sensitive DAMP once released in the extracellular space by immune activated, necrotic, and cancer cells. The fully reduced form is the only able to synergize with CXCL12, while reactive oxygen species lead to the formation of a disulfide bond between two cysteine, rendering the alarmin selective for TLR4 and mediating the production of pro-inflammatory chemokines and cytokines (34).

The role and relevance of the CXCL12/HMGB1 heterocomplex has been established in a model of sterile inflammation (6). Recent evidence indicates that the presence and activity of the heterocomplex can be either beneficial in models of tissue regeneration (9, 10), or detrimental in patients with active Rheumatoid Arthritis (8). However, how G protein and β-arrestins contribute to the CXCR4 signaling induced by the CXCL12/HMGB1 heterocomplex in modulating cellular responses was not elucidated so far.

In the present work, we demonstrated that the CXCL12/HMGB1 heterocomplex acts as a balanced agonist on CXCR4, inducing the activation of both G protein and β-arrestin mediated pathways. Indeed, the heterocomplex requires primarily β-arrestin1 for the induction of actin polymerization, while both β-arrestin1 and β-arrestin2 are necessary for directional migration. The heterocomplex, compared to CXCL12 alone, does not induce CXCR4 internalization, but preserves CXCR4 on the plasma membrane, in a process that is β-arrestin2 dependent. The present data indicate that the heterocomplex, compared to CXCL12, differentially engages the β-arrestin proteins, promoting a prompt availability of CXCR4 on the cell surface, and allowing an enhanced response to the chemotactic cue.

## Materials and Methods

### Reagents

CXCL12 was chemically synthesized as previously described (35). Full length HMGB1 and HMGB1-Histidine-tagged were produced and stored in phosphate-buffered saline (PBS; D8537, Sigma Aldrich, Saint Louis, MO, United States) at the Institute of Research in Biomedicine Protein Facility (Bellinzona, Switzerland), or purchased (1690-HMB, R&D Systems, Minneapolis, MN, United States). The chosen sub-optimal concentrations of CXCL12 and the concentration of HMGB1 in each experiment were based on our previous findings (6).

### Cell Culture

Wild type HeLa cells (CCL2TM from American Type Culture Collection, Rockville, MD, United States) were cultured in Dulbecco’s modified Eagle medium (DMEM) containing 4.5 g/L of D-glucose, and glutaMAX (61965-026, GIBCO, ThermoFisher Scientific, Switzerland) supplemented with 10% heat-inactivated fetal bovine serum (16000-044, GIBCO, ThermoFisher Scientific, Switzerland), 1% penicillin-streptomycin (15070-063, GIBCO, ThermoFisher Scientific, Switzerland), and maintained under standard conditions (5% CO_2_, 95% O_2_, 37°C). β-arrestin1 KO, β-arrestin2 KO, and β-arrestin1/2 KO HeLa cells, were cultured in the same medium and under standard culture conditions as described for wt cells. Cells were detached using 10 mM ethylenediaminetetraacetic acid (EDTA)/PBS for 3 min under standard culture conditions.

### β-Arrestin1 and β-Arrestin2 Recruitment to CXCR4 (BRET Assay)

Hela wt cells were transfected with pcDNA3-CXCR4-EGFP, pcDNA3-CXCR4-HA, pcDNA3-CCR7-EGFP, pcDNA3-CCR7-HA, pcDNA3-β-arrestin1-nLuc, or pcDNA3-β-arrestin2-nLuc using the Neon Transfection System (ThermoFisher Scientific, Switzerland) according to the manufacturer’s protocol using the 100 μl Kit. In brief, 5 × 10^5^ cells were electroporated with 10 μg total plasmid DNA in a 1:1 ratio of receptor and β-arrestin before seeding of 2.5 × 10^5^ cells in DMEM (PAN-Biotech GmbH, Aidenbach, Germany) containing 20 % FCS (Lonza, Basel, Switzerland) allowing receptor expression for at least 36 h. For BRET experiments, transfected cells were washed with PBS (Chemie Brunschwig, Basel, Switzerland) containing 5 mM glucose (PBS-G) and detached using Cell Dissociation Buffer (GIBCO, ThermoFisher Scientific, Switzerland) before collecting them in DMEM. After washing with PBS-G, cells were loaded with 5 μM coelenterazine H (Biosynth, Thal, Switzerland) and distributed in a white 96-well half-area plate (Tecan, Männedorf, Switzerland). Basal luminescence was followed for 10 min on a Spark M10 multiplate reader (Tecan, Männedorf, Switzerland) measuring EGFP fluorescence (505–509 nm, 500 ms integration time) and luciferase bioluminescence (384–440 nm, 500 ms integration time) before stimulation with the indicated ligand for further 30 min. BRET ratio was then calculated based on EGFP fluorescence and luciferase luminescence and baseline-corrected for BRET ratios of cells co-transfected with receptor-HA and β-arrestin1-nLuc or β-arrestin2-nLuc, respectively.

### Generation of Knock-Out Cells for the Different β-Arrestin Isoforms by CRISPR/Cas9 Genome Editing

β-arrestin1 KO, β-arrestin2 KO, and β-arrestin1/2 KO HeLa cells were generated by CRISPR/Cas9 using the following commercial plasmids: β-Arrestin1 CRISPR/Cas9 KO Plasmid (h; sc-400642), β Arrestin1 HDR Plasmid (h; sc-400642-HDR) selection plasmid for β-arrestin1 KO cells and for β-arrestin1/2 double KO generation, and β-Arrestin2 CRISPR/Cas9 KO Plasmid (h; sc-416686) for β-arrestin2 KO cells generation (all from Santa Cruz Biotechnology, Inc., Santa Cruz, CA, United States). Cells were transfected using Lipofectamine LTX reagent (15338-030, Invitrogen, Waltham, MA, United States), according to manufacturer’s instructions for 48 h, and GFP^+^/RFP^+^ double positive cells or GFP^+^ single positive, in the case of β-arrestin2 KO cells generation, were sorted with BD Aria Sorter (BD Bioscience, San Jose, CA, United States), and plated in a 48 wells plate. In the case of β-arrestin1 and β-arrestin1/2 KO cells, selection was obtained with 1 μg/mL puromycin (P7255, Sigma Aldrich, Saint Louis, MO, United States) for 7 days, followed by single cell cloning. β-arrestin2 KO cell clones were selected by limiting dilution and staining β-arrestin2 levels using an anti-β-arrestin2 antibody (Santa Cruz Biotechnology, Inc, Santa Cruz, CA, United States), and samples were acquired by flow cytometry using the BD CANTO (BD Biosciences, San Jose, CA, United States).

In order to confirm correct editing, genomic DNA was extracted from HeLa wt and β-arrestins KO clones using the DNeasy Blood & Tissue Kit (69504, QIAGEN, Hilden, Germany), according to manufacturer’s instructions. Amplification of DNA genomic products and Sanger genomic sequencing was performed by Microsynth AG (Balgach, Switzerland).

Further validation was performed by PCR analysis. RNA was extracted using TRIzol reagent (15596026, Invitrogen, Waltham, MA, United States), following manufacturer’s instructions. cDNA were obtained from RNA samples by retro-transcription using Moloney murine leukemia virus reverse transcriptase (28025-013, Invitrogen, Waltham, MA, United States) with 1 μg RNA per sample, and 250 ng of random primers, according to the manufacturer’s instructions. PCR was performed using Taq DNA polymerase enzyme (270799-61, GE Healthcare Life Sciences, Chicago, IL, United States) and 500 ng of cDNA for each sample, following manufacturer’s instructions. Forward and reverse primers were designed using the Primer3 software^1^ encompassing the RNA sequence region targeted by CRISPR/Cas9 in order to distinguish between wild-type and edited sequences. The sets of primers were synthetized by Microsynth AG (Balgach, Switzerland), and their sequence is indicated in Supplementary Table S1. Samples were prepared in loading dye (L3350, Us Biological, Salem, MA, United States) and separated in 1.8% agarose gels containing the DNA intercalant agent 1x RedSafe (21141, Intron Biotechnology, Gyeonggi-do, South Korea). Images were acquired using the Ingenius3 imager (Labgene Scientific SA, Châtel-Saint-Denis, Switzerland).

Lack of β-arrestin expression at the protein level was confirmed by Western blot analysis. Protein extraction was performed as follows: cells were washed with cold PBS and 1x sample buffer was added before cell scraping. Samples were sonicated, passed in syringes and boiled at 95°C for 5 min. Proteins were separated by sodium dodecyl sulfate-polyacrylamide gel electrophoresis (SDS-PAGE) in 12% acrylamide gel. The proteins were blotted onto a PVDF filter Immobilion-P (IPVH304F0, Millipore, Billerica, Massachusetts, MA, United States) with a standard semi-dry apparatus (ThermoFisher Scientific, Waltham, MA, United States). Blocking was performed for 1 h at room temperature in 5% milk/TBS-T, and the following primary antibodies, incubated at 4°C, overnight, were used for protein detection: rabbit anti-human β-arrestin1 (clone D7Z3W, 30036s, Cell signaling, Danvers, MA, United States), mouse anti-human β-arrestin2 (clone D-5, sc-166935, Santa Cruz Biotechnology, Inc., Santa Cruz, CA, United States), both diluted 1:500 in 5% BSA/TBS-T, and mouse anti-human GAPDH diluted 1:5000 in 5% milk/TBS-T. Membranes were incubated for 1 h at room temperature with the secondary anti-rabbit horseradish peroxidase (HRP)-conjugate antibody (7074P2, Cell signaling, Danvers, MA, United States), and anti-mouse Goat HRP-conjugate antibody (1706516, Bio-Rad, Hercules, CA, United States), diluted to 1:5000 in 5% milk/TBS-T. Super Signal (34077, ThemoFischer Scientific, Waltham, MA, United States) was used as luminol-based enhanced chemiluminescent substrate for detecting HRP on the immunoblots. Chemiluminescence reaction was detected using the Amersham^TM^ Imager 680 (GE Healthcare Life Sciences, Chicago, IL, United States).

### Actin Polymerization

HeLa wt and β-arrestins KO cells were grown on poly-D-lysine-coated dishes (P35GC-0-14C, MatTek Corporation, Ashland, MA, United States) for 24 h. After washing with PBS, cells were stimulated with increasing concentrations of CXCL12, in the presence or absence of 300 nM HMGB1, for 15 s under standard culture conditions. Cells were fixed in 4% PFA/PBS for 12 min on ice, and permeabilized with 0.01% Triton-X-100 for 2 min on ice. Filamentous actin was stained using 4 μg/mL Phalloidin-FITC (P5282, Sigma Aldrich, Saint Louis, MO, United States) for 30 min at room temperature in the dark. Nuclei were stained using DAPI for 2 min at room temperature in the dark. Samples were analyzed by confocal microscopy (Leica SP5, Heerbrugg, Switzerland) at 63X magnification. Mean fluorescence intensity (MFI) specific for filamentous-actin (F-actin) was quantified using the open-source ImageJ software. Relative MFI (rMFI) was calculated by dividing the MFI value of each cell for the mean MFI of the matched unstimulated condition. Values were plotted using the GraphPad Prism 7 (GraphPad software).

### Cell Migration

Cell migration in wt and β-arrestins KO HeLa cells was measured using the Ibidi μ-Slide chemotaxis system (80326, Martinsried, Germany). 1.8 × 10^4^ cells were diluted in DMEM containing 1% FBS and 20 mM Hepes (chemotaxis medium), and seeded in the channel of the chemotaxis slide according to the manufacturer’s instructions, and cultured for 6 h. The reservoirs were filled with chemotaxis medium and chemoattractant were applied into the right reservoir according to the manufacturer’s protocol. Cell migration induced by 10 nM CXCL12, in the presence or absence of 300 nM HMGB1, by 100 nM CXCL12, or by 300 nM HMGB1, was assessed. For experiments with the CXCR4 inhibitor AMD3100 (A5602, Sigma Aldrich, Saint Louis, MO, United States) both reservoirs were filled with the inhibitor, used at the final concentration of 1 μM, and chemotaxis was performed toward a CXCL12/HMGB1 gradient (10 nM CXCL12 and 300 nM HMGB1). For experiments with *Bordetella pertussis* toxin (PTX; P7208, Sigma Aldrich, Saint Louis, MO, United States), cells were pre-treated with 1 μg/ml PTX for 30 min before assessing migration.

**FIGURE 1:**
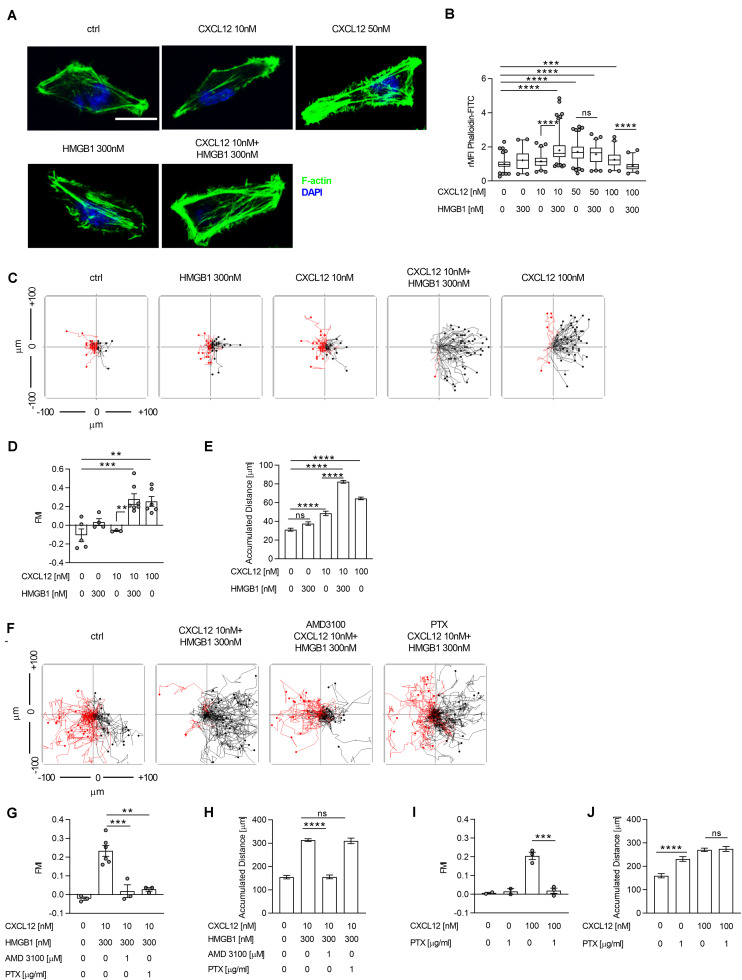
The CXCL12/HMGB1 heterocomplex enhances actin polymerization and chemotaxis via CXCR4 in HeLa cells. **(A)** Actin polymerization in HeLa cells stimulated for 15 s with the indicated concentrations of CXCL12 and/or HMGB1: F-actin (Phalloidin-FITC, green), and nuclei (DAPI, blue). Representative images, of at least 3 independent experiments, are shown. Scale bar represents 9 μm **(B)** Quantitative evaluation of the Phalloidin-FITC mean fluorescence intensity for each experimental condition. Relative mean fluorescence intensity (rMFI) was calculated measuring values specific for F-actin, and normalizing to the mean MFI of the matched unstimulated condition. Data are shown as box and whiskers (2.5–97.5 percentile; “+” mean) of at least 3 independent experiments analyzed with the ImageJ software. For each condition (left to right), the following number of cells were analyzed: *n* = 209; 104; 132; 243; 226; 144; 102; 92. **(C)** Cell migration assay. HeLa cells were allowed to migrate in response to the indicated concentrations of CXCL12 and/or HMGB1 for 18 h. Representative spider plots, showing the trajectories of at least 50 single tracked cells migrating following (black) or moving in the opposite direction (red) of the gradient, are shown. Black and red dots in the plots represent the final position of each single tracked cell. **(D)** Quantitative evaluation of the Forward Migration Index (FMI) is shown. Mean ± SEM of at least 3 independent experiments, tracking at least 50 cells. **(E)** Quantitative evaluation of the accumulated distance from the experiments shown in **(D)**. Pooled mean ± SEM of *n* = 250; 201; 151; 351; 299 cells analyzed. **(F)** Cell migration assay. HeLa cells were allowed to migrate in response to the CXCL12/HMGB1 heterocomplex for 18 h in the presence or absence of the CXCR4 inhibitor AMD3100, or of *Pertussis toxin* (PTX). Representative spider plots are shown. **(G–J)** Quantitative evaluation of FMI **(G,I)**, and accumulated distance **(H,J)** are shown. **(G,I)** Mean ± SEM of at least 2 independent experiments, tracking at least 50 cells. **(H,J)** Pooled mean ± SEM of *n* = 131; 260; 150; 125 **(H)**, and *n* = 100; 100; 141; 137 **(J)** cells analyzed. **p < 0.01, ***p < 0.001, ****p < 0.001, or ns (not significant), using one-way ANOVA followed by Tukey’s multiple comparison test.

Phase contrast images were recorded for 18 h with a time laps of 15 min using a 4X objective. Experiments presented in Figures 1C–E were performed at the Medical College of Wisconsin (Milwaukee, WI, United States) using an IX83 inverted microscope (Olympus, Waltham, MA, United States) equipped with an incubation system (Tokai Hit, Japan) set to 5% CO2, 37°C. Experiments presented in Figures 1F–J and in Figure 4 were performed at Institute of Research in Biomedicine (Bellinzona, Switzerland) using the ImageXpress Micro 4 Imager (Molecular Devices, San Jose, CA, United States) equipped with an incubation system set to 5% CO2, 37°C. Single cell tracking was performed selecting the center of mass in each frame using the manual tracking plug-in tool for the software ImageJ. Approximately 50 cells were tracked for each independent experiment. Spider plots, representing the trajectories of the tracked cells, forward migration index (FMI), and accumulated distance values were obtained using the chemotaxis and migration plug-in tool (https://ibidi.com/chemotaxis-analysis/171-chemotaxis-and-migration-tool.html, Ibidi, Martinsried, Germany). The FMI represents the efficiency of the forward migration of cells. The larger the FMI, the stronger the chemotactic effect is toward the stimulus. The accumulated distance represents the length of the cell path, from the starting point to the endpoint.

### CXCR4 Internalization and Recycling Assessed by Confocal Microscopy

HeLa wt and β-arrestins KO cells were grown on poly-D-lysine-coated dishes (P35GC-0-14C, MatTek Corporation, Ashland, MA, United States) for 24 h under culture conditions. Cells were then transfected for 24 h using Lipofectamine LTX reagent (15338-030, Invitrogen, Waltham, MA, United States) with a plasmid expressing a CXCR4-acyl carrier protein (ACP) tagged, according to manufacturer’s instructions. This construct enables the specific labeling of the receptor in order to follow its trafficking within the cells, and CXCR4 cycling on the plasma membrane (36, 37). After transfection, the medium was replaced with fresh medium and cells were cultured for 1 h under standard conditions, to allow cell recovery after transfection. For internalization experiments, CXCR4 labeling was performed before stimulation incubating cells with 40 μM ACP-synthase enzyme (P9301S), 10 mM MgCl_2_, and CoA-647 dye (S9350S), all purchased from New England BioLabs (Ipswich, MA, United States), in complete medium for 10 min at room temperature in the dark. After washing with PBS, cells were stimulated with complete medium or 300 nM HMGB1 as controls, and 10 nM CXCL12 in the presence or absence of 300 nM HMGB1 for 30 min under standard culture conditions. Cells were fixed in 4% PFA/PBS for 10 min at room temperature, washed two times in PBS, and nuclei stained with DAPI 2 min at room temperature. CXCR4 relative intensity at the surface or in the intracellular compartment was determined by dividing their specific CXCR4-ACP values for the total expression of the receptor in each cell. Data were then normalized to the relative expression of CXCR4 in stimulated cells over the matched unstimulated control.

For CXCR4 recycling experiments, stimulation was performed as described above and, after removal of the chemoattractant, cells were cultured under standard conditions for 1 h. After 1 h, CXCR4 labeling was performed as described before for the internalization experiments, and cells fixed in 4% PFA/PBS 10 min at room temperature. Samples were analyzed by confocal microscopy (Leica SP5, Heerbrugg, Switzerland) at 63X magnification. MFI quantification of the 647 channel, specific for CXCR4 staining, was quantified using the open-source ImageJ software. MFI values were plotted using the GraphPad Prism 7 software.

### Statistical Analysis

The statistical significance between two groups was assessed using unpaired, two tailed *t*-test. Statistical significance between more than two groups was calculated by using one-way or two-way ANOVA followed by Tukey’s multiple comparison test, or by Dunnett’s multiple comparison test, as appropriate. A *p* value below 0.05 was considered as significant.

## Results

### The CXCL12/HMGB1 Heterocomplex Enhances Filamentous Actin Polymerization and Chemotaxis

To study the signaling induced by the CXCL12/HMGB1 heterocomplex, we took advantage of HeLa cells, which express high levels of CXCR4, the selective receptor for the heterocomplex (6, 7), while the receptor TLR-4, which binds to oxidized HMGB1 alone and not to the heterocomplex (7) is expressed at very low levels on the cell surface (Supplementary Figure S1).

The ability of HeLa cells to rearrange the cytoskeleton, after stimulation, was assessed by confocal microscopy after staining for filamentous actin (F-actin), as index of the CXCR4-mediated actin polymerization. The maximal effect on actin polymerization was observed stimulating cells with 50 nM CXCL12, whereas no response was induced with a sub-optimal concentration of 10 nM CXCL12 (Figures 1A,B). Notably, stimulation of cells with the CXCL12/HMGB1 heterocomplex, formed with the sub-optimal CXCL12 dose and 300 nM HMGB1, led to an actin rearrangement comparable to the one obtained by stimulating the cells with the optimal dose of the chemokine (50 nM; Figures 1A,B). Stimulation of cells with the CXCL12/HMGB1 heterocomplex, formed with the optimal CXCL12 concentration or higher (50 nM and 100 nM), did not result in a further increase in actin polymerization (Figure 1B). Cytoskeleton rearrangement is essential to sustain cell migration in presence of a chemotactic gradient; therefore, chemotaxis induced by the CXCL12/HMGB1 heterocomplex was assessed using the Ibidi μ-slide system, as previously described (26). In agreement with the above results, the sub-optimal CXCL12 concentration did not induced cell migration. As demonstrated by the FMI, chemotaxis induced by 10 nM CXCL12 was negligible, however, the same chemokine concentration in a CXCL12/HMGB1 heterocomplex induced comparable directional migration as the optimal CXCL12 concentration (100 nM; Figures 1C,D). Cell migration induced by the CXCL12/HMGB1 heterocomplex was accompanied by a significant increase in the accumulated distance, compared to cells stimulated with a sub-optimal CXCL12 dose (Figure 1E). As control, when CXCL12 was added to both reservoirs of the chemotaxis system, no directional migration was observed (Supplementary Figures S2A,B). As shown in other cell types (6), we confirmed that the chemotaxis induced by the heterocomplex is CXCR4 dependent, since AMD3100, a CXCR4 inhibitor, completely abrogated cell migration (Figures 1F,G). Of note, pretreatment of HeLa cells with the Gα_i_ subunit inhibitor *Bordetella pertussis* toxin (PTX) completely abrogated the heterocomplex-mediated chemotaxis, indicating that the G protein signaling is required for the heterocomplex promoted cell migration (Figures 1F,G). Interestingly, as shown by the accumulated distance, PTX treatment interfered with cell directionality, but not with chemokinesis (Figures 1H–J). Uncoupling G proteins from the receptor resulted in an increased cell motility also in the absence of any chemoattractant (Figure 1J).

These results indicate that the G protein signaling induced by the heterocomplex is required to sustain an efficient directional cell migration, while a G protein-independent signaling event contributes to sustain chemokinesis.

### The CXCL12/HMGB1 Heterocomplex Recruits β-Arrestins to CXCR4

**FIGURE 2:**
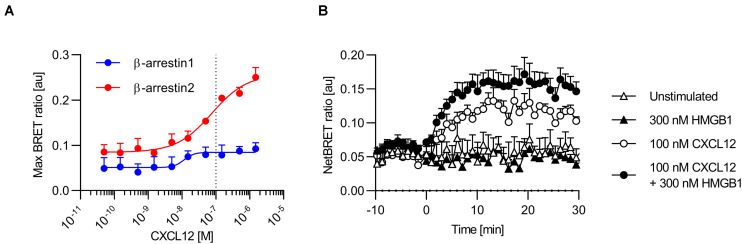
CXCL12 and the CXCL12/HMGB1 heterocomplex recruit β-arrestins to CXCR4. **(A)** HeLa cells co-expressing CXCR4-EGFP and β-arrestin1-nLuc or β-arrestin2-nLuc were stimulated with graded concentrations of CXCL12. Chemokine-mediated β-arrestin recruitment to CXCR4 was determined by BRET. Mean ± SEM of 3 independent experiments. Dotted line represents the sub-optimal CXCL12 concentration for β-arrestin2 recruitment to CXCR4 **(B)** HeLa cells co-expressing CXCR4-EGFP and β-arrestin2-nLuc were stimulated at time point 0 with either 100 nM CXCL12, 300 nM HMGB1, or with the heterocomplex (100 nM CXCL12/300 nM HMGB1). Representative NetBRET ratios (Mean ± SEM) of duplicate values from one out of three independent experiments are shown.

β-arrestin proteins play a crucial role in promoting CXCL12-mediated signaling and in sustaining chemotaxis (22, 26). We used BRET assays to determine CXCL12-mediated recruitment of β-arrestins to CXCR4. To this end, we co-expressed β-arrestin1 and β-arrestin2 tagged at the C-terminus with the nanoluciferase nLuc and CXCR4 labeled with EGFP at the C-terminus in HeLa cells, and stimulated them with graded concentrations of CXCL12 (38). In line with published data (23, 29), CXCL12 efficiently recruited β-arrestin2 to CXCR4 in a concentration dependent manner (Figure 2A). CXCL12 also recruited β-arrestin1 to CXCR4 although much less efficiently (Figure 2A), which confirms a previous study in which the luciferase was fused to the C-terminus of CXCR4 and the fluorophore to the N-termini of arrestins (29). Notably, the expression of CXCR4 measured as total fluorescence intensity of CXCR4-EGFP was comparable in cells co-expressing either β-arrestin1-nLuc or β-arrestin2-nLuc (Supplementary Figure S3A). In addition, the luciferase turnover was similar in cells expressing either β-arrestin1-nLuc or β-arrestin2-nLuc (Supplementary Figure S3B), demonstrating that the reduced efficiency of β-arrestin1 recruitment to CXCR4 is not due to altered expression levels of the chemokine receptor or the β-arrestins. Moreover, β-arrestin1-nLuc and β-arrestin2-nLuc were both recruited to CCR7 upon agonist stimulation (Supplementary Figure S3C), revealing that both β-arrestin-nLuc constructs are functional. We then used 100 nM CXCL12, a sub-optimal concentration for β-arrestin2 to CXCR4 in this assay, to assess the activity of the heterocomplex. In presence of the CXCL12/HMGB1 heterocomplex, we observed an increase of β-arrestin2 recruitment to CXCR4, compared to CXCL12 alone, even if not statistically significant (Figure 2B). We found no recruitment of β-arrestin2 to CXCR4 by stimulating cells solely with HMGB1 (Figure 2B). As the signal for CXCL12-mediated β-arrestin1 recruitment to CXCR4 was weak and rapidly reached a plateau, we could not reliably determine the synergistic effect of the heterocomplex on β-arrestin1 recruitment. However, our data provide evidence that β-arrestins are recruited to CXCR4 upon agonist stimulation.

### The CXCL12/HMGB1 Heterocomplex-Mediated Actin Polymerization Is β-Arrestin1/2 Dependent

β-arrestin proteins are key players in CXCL12-induced responses via CXCR4, but so far no data were available on their roles in promoting cytoskeleton remodeling after receptor triggering with the CXCL12/HMGB1 heterocomplex.

In order to dissect the role of β-arrestins in actin polymerization and chemotaxis, we generated β-arrestin1, β-arrestin2, and β-arrestin1/2 KO HeLa cells using CRISPR/Cas9 technology. The gene editing strategy used was specific for each individual β-arrestin isoform as assessed by PCR (Supplementary Figure S4A), and by genomic sequencing of the two β-arrestin exons targeted by CRISPR/Cas9 (Table 1). These results were further confirmed by western blot analysis (Supplementary Figures S4B,C). Knocking-out one of the two β-arrestin isoforms did not alter the expression of the other β-arrestin isoform, nor CXCR4 surface levels (Supplementary Figures S4B–D).

**TABLE 1:** CRISPR/Cas9 β-arrestin gene editing obtained by sequencing analysis.

**Sample**	**β1 Exon 3**	**β1 Exon 5**	**β2 Exon 2**	**β2 Exon 3**
wt	wt/wt	wt/wt	wt/wt	wt/wt
β1 KO	HDR/HDR	HDR/edited	wt/wt	wt/wt
β2 KO	wt/wt	wt/wt	Edited/edited	Edited/edited
β1/2 KO	HDR/HDR	Edited/edited	Edited/edited	Edited/edited

*Gene editing of the β-arrestin1 exon 3, and 5, and β-arrestin2 exon 2, and 3, targeted by CRISPR/Cas9 plasmids, performed on genomic DNA from wt, or β-arrestins KO HeLa cells. HDR represents the sequence encoding for the puromycin resistance.*

**FIGURE 3:**
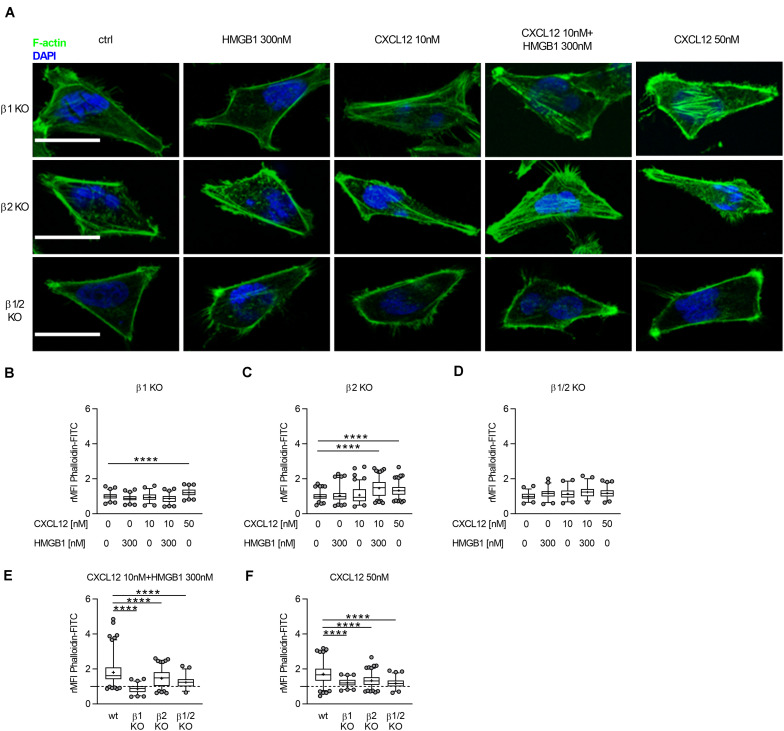
The CXCL12/HMGB1 heterocomplex-mediated actin polymerization requires β-arrestin1 and β-arrestin2. **(A)** Actin polymerization in HeLa β-arrestin KO cells stimulated for 15 s with the indicated concentrations of CXCL12 and/or HMGB1: F-actin (Phalloidin-FITC, green), and nuclei (DAPI, blue). Representative images, of at least 3 independent experiments, are shown. Scale bar represents 9 μm. **(B–F)** Relative mean fluorescence intensity (rMFI) was calculated measuring values specific for F–actin, and normalizing to the mean MFI of the matched unstimulated condition. Data are shown as box and whiskers (2.5–97.5 percentile; “+” mean) of at least 3 independent experiments analyzed with the ImageJ software. The following number (*n*) of cells were analyzed: *n* = 131; 142; 109; 145; 132 **(B)**; *n* = 217; 184; 221; 284; 250 **(C)**; *n* = 111; 97; 112; 105; 144 **(D)**; *n* = 243; 145; 284; 105 **(E)**; and *n* = 226; 132; 250; 144 **(F)**. ****p < 0.001 using one-way ANOVA followed by Dunnett’s multiple comparison test.

We measured, by confocal microscopy, the ability of β-arrestin1, β-arrestin2, or β-arrestin1/2 KO HeLa cells to polymerize actin in response to CXCL12 or the heterocomplex (Figure 3A). Actin rearrangement was observed in β-arrestin1, and β-arrestin2 KO HeLa cells, but not in β-arrestin1/2 KO HeLa cells in response to 50 nM CXCL12. Stimulation of cells with the CXCL12/HMGB1 heterocomplex induced actin polymerization only in β-arrestin2 KO HeLa cells (Figures 3B–D). Of note, the actin polymerization induced by both the heterocomplex and the optimal concentration of CXCL12 was significantly decreased in β-arrestins KO compared to wt HeLa cells, suggesting a contribution of both β-arrestins in this process (Figures 3E,F).

These results indicate that the CXCL12/HMGB1 heterocomplex-mediated actin polymerization depends on both β-arrestin isoforms, with a prominent role exerted by β-arrestin1. In contrast, β-arrestin1 or β-arrestin2 individually contribute to cytoskeleton remodeling in response to CXCL12 alone. Notably, actin polymerization in response to CXCL12 or the heterocomplex is abrogated in cells lacking both β-arrestins.

### The CXCL12/HMGB1 Heterocomplex-Mediated Chemotaxis Is Both β-Arrestin1 and β-Arrestin2 Dependent

**FIGURE 4:**
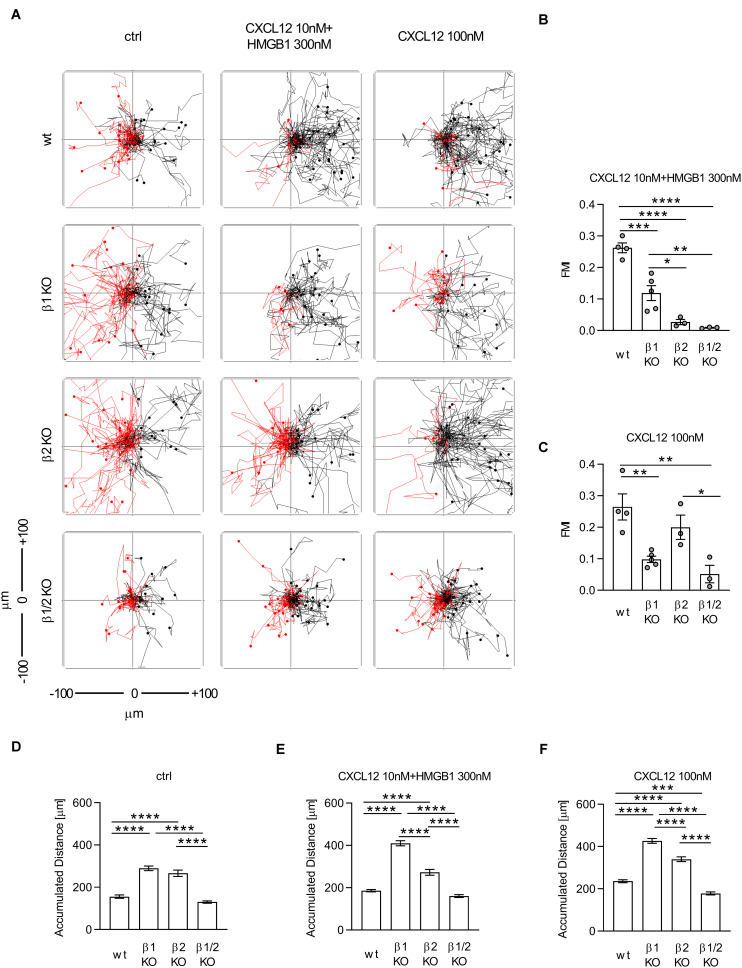
The CXCL12/HMGB1 heterocomplex-mediated chemotaxis requires both β-arrestin1 and β-arrestin2. **(A)** Cell migration assay. Wt and β-arrestin KO HeLa cells were allowed to migrate in response to the indicated concentrations of CXCL12 and/or HMGB1 for 18 h. One representative spider plot, out of three independent experiment performed, is shown for each experimental condition. Trajectories of at least 50 single tracked cells migrating following (black) or moving in the opposite direction (red) of the gradient, are shown. Black and red dots in the plots represent the final position of each single tracked cell. **(B,C)** Quantitative evaluation of the Forward Migration Index (FMI) after stimulation with the CXCL12/HMGB1 heterocomplex **(B)** or with 100 nM CXCL12 **(C)** in wt and β-arrestin KO HeLa cells. Mean ± SEM of at least 3 independent experiments, tracking *n* = 25÷50 cells. **(D–F)** Quantitative evaluation of the accumulated distance after stimulation with the vehicle only **(D)**, the CXCL12/HMGB1 heterocomplex **(E)**, or with 100 nM CXCL12 **(F)** in wt and β-arrestin KO HeLa cells. Data are shown as pooled mean ± SEM of *n* = 181; 150; 81; 136 **(D)**; *n* = 178; 230; 108; 150 **(E)**, *n* = 179; 230; 118; 150 **(F)** cells analyzed. *p < 0.05, **p < 0.01, ***p < 0.001, and ****p < 0.0001, using one-way ANOVA followed by Tukey’s multiple comparison test.

It has been previously demonstrated that the chemotaxis induced by CXCL12 in HeLa cells is β-arrestin1 and β-arrestin2 dependent (22, 26). Therefore, we performed cell migration experiments in order to dissect which β-arrestin isoform was required to sustain the heterocomplex-mediated chemotaxis. We found that β-arrestin1 and β-arrestin2 were both essential at different degrees to promote chemotaxis induced by the CXCL12/HMGB1 heterocomplex, since a significant decrease in directional cell migration was observed in β-arrestin1, β-arrestin2, and β-arrestin1/2 KO cells (Figures 4A,B).

In agreement with previous findings (26), we observed that CXCL12-mediated migration was β-arrestin1 dependent. In contrast, we found that the lack of β-arrestin2 did not influence directional migration at optimal CXCL12 concentration (Figures 4A,C).

The impaired chemotaxis observed in β-arrestin1 KO cells stimulated with both the heterocomplex or CXCL12 alone, was not associated to a decrease in cell motility, as the measured accumulated distance of those cells was significantly higher, compared to wt cells, in any condition tested (Figures 4D–F).

### The CXCL12/HMGB1 Heterocomplex Prevents CXCR4 Internalization in a β-Arrestin2 Dependent Manner

CXCR4 desensitization and internalization are both essential for the regulation of CXCL12-mediated responses and are processes that require β-arrestins (39, 40). Upon internalization, CXCR4 accumulates on endosomes where it is sorted for degradation in lysosomes (33). We transfected wt, β-arrestin1, β-arrestin2, or β-arrestin1/2 KO HeLa cells with CXCR4 N-terminally ACP-tagged, which can be specifically labeled at the plasma membrane (36), allowing to study receptor trafficking within the cell by confocal microscopy.

**FIGURE 5:**
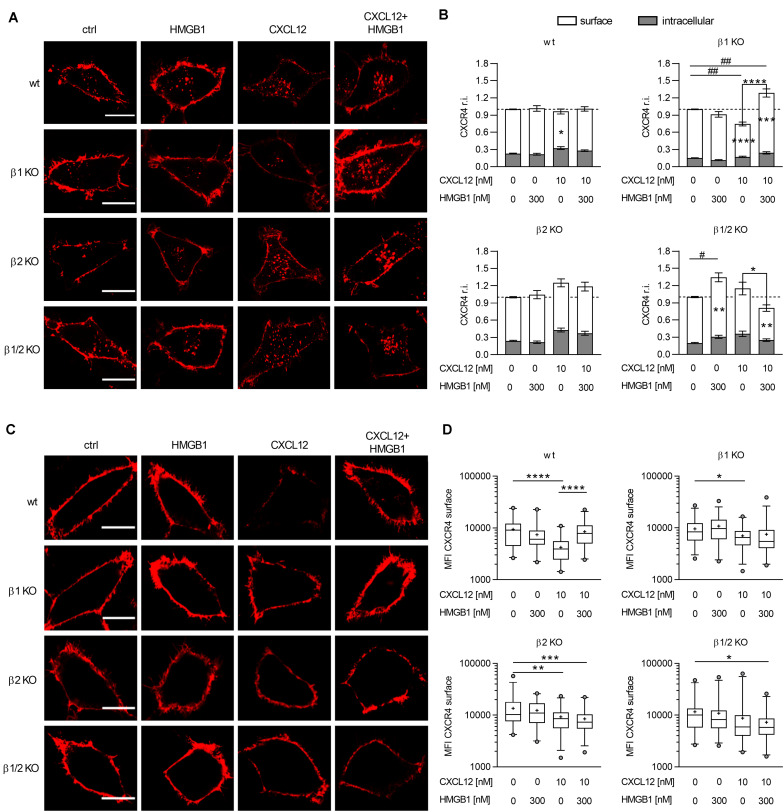
The CXCL12/HMGB1 heterocomplex preserves CXCR4 on the cell membrane in a β-arrestin2 dependent manner. **(A)** CXCR4 trafficking in wt or β-arrestin KO HeLa cells, stimulated for 30 min with the indicated concentrations of CXCL12 and/or HMGB1: CXCR4-ACP (red). Representative images, of at least 3 independent experiments, are shown. Scale bar represents 6 μm. **(B)** Quantitative evaluation of CXCR4-ACP expression for each experimental condition. CXCR4 relative intensity (r.i.) on the cell surface or in the intracellular compartment was determined by dividing their specific CXCR4-ACP values for the total expression of the receptor in each cell. Data were then normalized to the relative expression of CXCR4 in stimulated cells over the matched unstimulated control. White bars represent surface CXCR4 expression, gray bars represent intracellular CXCR4 expression. Data are shown as mean ± SEM of at least 3 independent experiments analyzed with the ImageJ software. The following number of cells were analyzed (left to right): *n* = 70; 76; 77; 93 (wt); *n* = 56; 49; 59; 49 (β1 KO); *n* = 36; 38; 55; 65 (β2 KO); and *n* = 44; 45; 33; 33 (β1/2 KO). *p < 0.05, **p < 0.01, ***p < 0.001, and ****p < 0.0001: two-way ANOVA, followed by Tukey’s multiple comparison test was used to determine differences in surface or intracellular CXCR4 r.i. among groups. ^#^*p* < 0.05, ^##^*p* < 0.01 one-way ANOVA, followed by Dunnett’s multiple comparison test was used to determine differences in total CXCR4 r.i. compared to unstimulated cells. **(C)** CXCR4 expression on the cell surface in wt or β-arrestin KO HeLa cells. HeLa cells overexpressing CXCR4-ACP were stimulated for 30 min with the indicated concentrations of CXCL12 and/or HMGB1 and then cultured for 1 h in fresh medium to allow CXCR4 recycling back to the cell surface: CXCR4-ACP (red). Representative images, of at least 3 independent experiments, are shown. Scale bar represents 6 μm. **(D)** Quantitative evaluation of CXCR4-ACP mean fluorescence intensity for each experimental condition. Data are shown as box and whiskers (2.5–97.5 percentile; “+” mean) of at least 3 independent experiments analyzed with the ImageJ software. The following number (*n*) of cells were analyzed: *n* = 45; 42; 52 (wt); *n* = 69; 64; 59; 73 (β1 KO); *n* = 62; 58; 67; 65 (β2 KO); and *n* = 53; 66; 62; 72 (β1/2 KO). *p < 0.05, **p < 0.01, ***p < 0.001, and ****p < 0.0001 using one-way ANOVA, followed by Tukey’s multiple comparison test.

When CXCR4-ACP wt cells were stimulated with the CXCL12/HMGB1 heterocomplex no significant differences were observed in the distribution of surface or intracellular CXCR4 compared to unstimulated cells. Stimulation with CXCL12 alone at 10 nM concentration, which does not activate the cytoskeleton machinery, induced a significant decrease in the surface levels of CXCR4, and a concomitant accumulation of the receptor in the intracellular compartment, which nevertheless did not reach significance (Figures 5A,B).

In β-arrestin1 KO cells we observed a different distribution in the surface and in the intracellular pool of CXCR4 compared to wt cells, with a higher percentage of CXCR4 present on the cell surface in β-arrestin1 KO cells (*p* < 0.0001, one-way ANOVA followed by Dunnett’s multiple comparison test). Stimulation with the CXCL12/HMGB1 heterocomplex induced a significant accumulation of CXCR4, which primarily localized on the cell surface, compared to unstimulated cells. Triggering of the receptor with CXCL12 alone resulted in a sharp decrease in the total expression of CXCR4, with significant lower levels of CXCR4 present on the cell surface, and no evident intracellular receptor accumulation compared to unstimulated cells. This resulted in a striking difference in CXCR4 expression on the cell surface, when comparing cells stimulated with the heterocomplex or the chemokine alone (Figures 5A,B).

Data obtained in β-arrestin2 KO cells showed no significant differences, compared to control, in CXCR4 total, surface, or intracellular levels upon stimulation with either the CXCL12/HMGB1 heterocomplex or CXCL12 alone (Figures 5A,B).

As for β-arrestin1 KO cells, in β-arrestin1/2 KO cells we observed a higher percentage of CXCR4 on the cell surface compared to wt cells (*p* = 0.0243, one-way ANOVA followed by Dunnett’s multiple comparison test). In β-arrestin1/2 KO cells only the stimulation with the heterocomplex induced a significant reduction in the surface expression of the receptor, even if stimulation with HMGB1 alone, which does not bind CXCR4, unexpectedly induced its accumulation (Figures 5A,B).

In summary, when cells are stimulated with CXCL12 alone, at doses that do not induce activation of the cytoskeleton machinery, CXCR4 internalization requires β-arrestin2, while in the absence of β-arrestin1 the receptor can be internalized, and is possibly promptly degraded. The CXCL12/HMGB1 heterocomplex does not promote CXCR4 internalization in CXCR4-ACP wt cells, indicating that upon heterocomplex stimulation the receptor remains available on the cell surface. Only in the absence of both β-arrestins the heterocomplex is able to induce a significant internalization, suggesting that in this condition a different mechanism can take place. In cells triggered with the heterocomplex, the only presence of β-arrestin2 in β-arrestin1 KO, leads to a significant accumulation of CXCR4 on the cell surface, suggesting that different stimuli dictate the role of β-arrestin2.

### The CXCL12/HMGB1 Heterocomplex Preserves CXCR4 on the Plasma Membrane in a β-Arrestin2 Dependent Manner

As CXCL12 stimulation promotes CXCR4 intracellular sorting to degradative lysosomes (39), we corroborated the data obtained on receptor internalization with the study of CXCR4 cycling to the plasma membrane upon removal of the stimulus. After stimulation for 30 min with the CXCL12/HMGB1 heterocomplex or with 10 nM CXCL12, which does not induce activation of the cytoskeleton machinery, cells were cultured in fresh medium allowing receptor cycling from the intracellular compartment, and then CXCR4 surface expression was detected by labeling the ACP tag.

CXCR4-ACP wt HeLa cells stimulated with the CXCL12/HMGB1 heterocomplex showed a higher expression level of CXCR4 on the plasma membrane compared to cells stimulated with CXCL12 alone, in line with the lack of internalization observed in the Figure 5B (Figures 5C,D). In β-arrestin2 and β-arrestin 1/2 KO cells, a significant lower expression of CXCR4 on the cell surface compared to control was observed, when cells were stimulated with the heterocomplex (Figures 5C,D).

Similar to published data, after stimulation with CXCL12 alone we did not observe receptor recycling to the plasma membrane in CXCR4-ACP wt HeLa cells, indicating that CXCR4 was targeted to degradation (32) (Figures 5C,D). In cells lacking β-arrestin1 or β-arrestin2, a significant decrease in the surface expression of the receptor was observed. Lack of both β-arrestin isoforms resulted in higher levels of CXCR4 expression on the cell surface, in line with the impaired internalization of the receptor observed, as in Figures 5B–D, and further sustaining the essential role of β-arrestins in CXCR4 degradation.

Overall, our results indicate that when cells are stimulated with the CXCL12/HMGB1 heterocomplex, the presence of β-arrestin2 is important to maintain the expression of the receptor on the cell surface. Indeed, in β-arrestin2 KO and in β-arrestin1/2 KO cells, the internalized receptor cannot recycle to the plasma membrane.

## Discussion

The ability of chemokines to form heterocomplexes with other chemokines or HMGB1 represents an additional level of regulation for shaping their function by enhancing their potency on their cognate receptor (5, 41, 42). In recent years, the relevance of the CXCL12/HMGB1 heterocomplex activity has been widely demonstrated in *in vivo* models of inflammation and tissue regeneration, and in human pathological conditions (6, 8–10, 42).

Our disclosure of the activity of the CXCL12/HMGB1 heterocomplex in Rheumatoid Arthritis (8), and the fact that it induces a different conformational change in CXCR4 dimers compared to stimulation with CXCL12 alone (6), prompted us to further investigate the downstream signaling events elicited by CXCR4 triggering.

In the current work, we demonstrated that the CXCL12/HMGB1 heterocomplex is a balanced agonist for CXCR4, which engages the β-arrestin proteins differently from CXCL12, promoting a prompt availability of CXCR4 on the cell surface, and enhancing directional cell migration.

**FIGURE 6:**
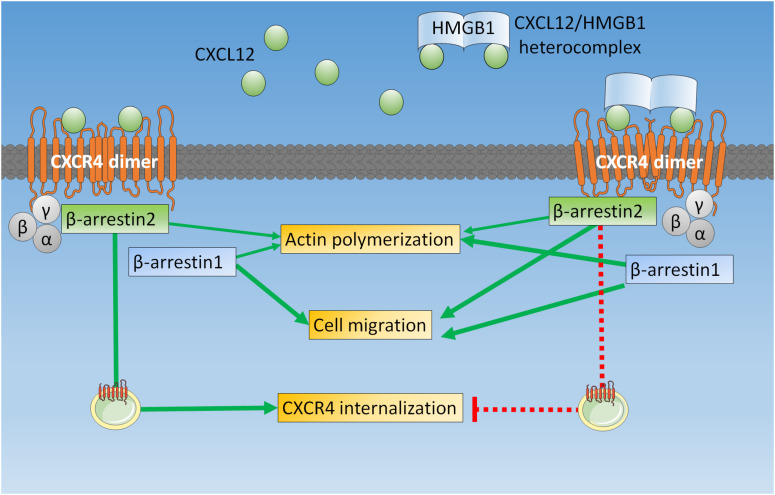
β-arrestins play different roles in mediating CXCR4 signaling upon triggering with CXCL12 or with the CXCL12/HMGB1 heterocomplex. In HeLa cells, when CXCR4 homodimers are triggered by CXCL12 alone, both β-arrestins contribute to actin polymerization, β-arrestin1 is required for cell migration, and β-arrestin2 allows receptor internalization and sorting for degradation. On the contrary, the CXCL12/HMGB1 heterocomplex-mediated actin polymerization is primarily β-arrestin1 dependent, while chemotaxis requires both β-arrestin1 and β-arrestin2. Triggering of CXCR4 homodimers with the CXCL12/HMGB1 heterocomplex leads to receptor retention on the cell surface, which depends on β-arrestin2.

The enhanced cytoskeleton remodeling and chemotaxis we observed in HeLa cells confirm that the heterocomplex can act on a variety of cell types (6, 8, 10), including tumor cells. We further dissect these findings in β-arrestin KO cells, showing that upon stimulation with the heterocomplex actin polymerization and cell migration requires the presence of both β-arrestin isoforms: β-arrestin1 is primarily involved in cytoskeleton remodeling; while β-arrestin2 has a prominent role in sustaining directional migration, maintaining the receptor available on the cell surface (Figure 6).

Both AMD3100 and PTX completely block directional cell migration induced by the CXCL12/HMGB1 heterocomplex, as previously shown in murine cells (6). While AMD3100 inhibits the activation of CXCR4, preventing the signaling through both β-arrestins and G proteins, PTX, blocks only Gα_*i*_ proteins, and still allows the β-arrestins to be engaged by CXCR4 (43, 44). The maintenance of cell motility, and the loss of directional migration that we have observed in cells stimulated with the heterocomplex and treated with PTX, indicate that β-arrestins and Gα_*i*_ proteins are both necessary for the activity of the heterocomplex: the first for promoting cell movement, and the latter for an efficient directionality. This requirement is not exclusively associated to the activity of the CXCL12/HMGB1 heterocomplex, but is shared with the activity of other chemokines and on different cell types (45, 46).

Our data on the impairment in the ability to rearrange the cytoskeleton observed in β-arrestins KO cells indicates that CXCR4-mediated actin polymerization requires the activation of both G-proteins-dependent (47, 48), and β-arrestins-dependent pathways. Indeed, β-arrestins by acting as scaffold for several proteins, mediate GPCRs-induced cytoskeleton remodeling (49). Of note, the heterocomplex and CXCL12 alone differently engage the two β-arrestin isoforms. When cells are triggered with CXCL12 alone, each β-arrestin isoform partially sustains rearrangement of the cytoskeleton. β-arrestin1 is essential for cytoskeleton rearrangement when cells are triggered with the heterocomplex, while lack of β-arrestin2 can be marginally overcome by the presence of β-arrestin1.

We demonstrated that, upon stimulation with CXCL12, both β-arrestin1 and β-arrestin2 are recruited to CXCR4, thus confirming a direct interaction of both β-arrestins with the phosphorylated CXCR4 (29). Our data show that β-arrestin2 recruitment to CXCR4 is enhanced in the presence of the CXCL12/HMGB1 heterocomplex and that both β-arrestin1 and β-arrestin2 are essential for the heterocomplex-mediated migration, while CXCL12-induced migration is β-arrestin1 dependent. β-arrestin2 has been shown in different reports to be essential for promoting CXCL12-induced chemotaxis (22, 23). In our experimental conditions, at 100 nM concentration of CXCL12, we observed that β-arrestin2 is not essential to induce HeLa cell migration, even though it is recruited to the receptor. These results might depend on the concentration of chemokine used, on the time of stimulation, the cell type, and the different type of assays performed which, in our case, allow the study of chemotaxis in adherent cells in the presence of a gradient. Indeed, in this experimental setting the contribution of adhesion molecules and focal adhesions and their influence in the process of cell polarization and directionality play a fundamental role (26). In fact, also in the paper by Sun et al., which shows stimulation with various CXCL12 concentrations, at 100 nM the differences in cell migration between wt HeLa cells, and cells silenced for β-arrestin2 are not significant (22).

CXCR4 regulation of internalization and trafficking is a crucial step in order to shape its induced responses. We found that the CXCL12/HMGB1 heterocomplex preserves CXCR4 on the plasma membrane compared to CXCL12 alone. Of note, we found that in cells lacking β-arrestin1 and triggered by the chemokine alone, CXCR4 is efficiently internalized, and possibly promptly degraded. These data indicate that β-arrestin1 is not required for CXCL12-induced CXCR4 internalization. Indeed, a prominent role for β-arrestin2 in the regulation of CXCR4 internalization has been previously described (50). Moreover, an AP-2-dependent/β-arrestin1-independent mechanism of internalization has been proposed for CXCR4 (51), and the disruption of the β-arrestin1/STAM-1 complex (26), due to lack of β-arrestin1, could be responsible of an accelerated CXCR4 degradation. When β-arrestin1 KO cells were stimulated with the CXCL12/HMGB1 heterocomplex, we did not observe CXCR4 internalization/degradation, but rather an accumulation of the receptor on the cell surface, indicating a different contribution for β-arrestin1 in the regulation of receptor trafficking between cell compartments. Most importantly, our data imply that β-arrestin2, does not sustain CXCR4 internalization when the receptor is triggered by the heterocomplex. We suggest that triggering of the receptor with CXCL12 alone or the heterocomplex induces the recruitment of different molecules, and that β-arrestins might be essential players in scaffolding these machineries. Moreover, several GRKs can phosphorylate the receptor upon activation and lead to the recruitment in its proximity of molecules that can determine the activation of downstream signals (52). Whether the different conformational changes of CXCR4 homodimers induced by the heterocomplex or the chemokine alone (6) lead to differential pattern of receptor phosphorylation or to the recruitment of additional molecules is still to be defined.

It is known that CXCL12 stimulation promotes AIP4-mediated CXCR4 ubiquitination at the plasma membrane, a crucial event for the delivery of the receptor to the endosomal compartments (32, 33, 53). The ubiquitinated CXCR4 undergoes sorting, through β-arrestin1 interaction with ESCRT machinery, for degradation into lysosomes (33). The CXCL12/HMGB1 heterocomplex might alter CXCR4 pattern of ubiquitination or might activate deubiquitin enzymes (54, 55) that, by removing ubiquitin moieties from CXCR4, could prevent CXCR4 sorting to lysosome and preserve the receptor on the plasma membrane. These data support the hypothesis that the increased receptor availability on the cell surface accounts for a sustained response to the chemotactic cue induced by the heterocomplex.

We have demonstrated that β-arrestins contribute differently to the activity of the heterocomplex and of CXCL12 alone. However, the different contribution of the β-arrestin isoforms to CXCR4 signaling responses induced by the heterocomplex might be cell type specific, thus resulting in selective effects. β-arrestins have been described to exert different activities in tumors, either promoting or preventing cancer cell migration and metastasis (56–60). Although CXCL12 and HMGB1 are overexpressed by different tumors and are associated with tumor progression and metastasis (19, 61), the functions of the CXCL12/HMGB1 heterocomplex in cancer remain to be elucidated (62). We should take into account that in different cell types, which could either express different levels of CXCR4 or of the two β-arrestin isoforms (15), the activity of the heterocomplex might result in opposite functions.

These data unveil the signaling induced by the heterocomplex in view of identifying biased antagonists targeting the heterocomplex selective pathways, leaving unaltered the beneficial activity of CXCL12, in order to improve therapies in pathologic conditions. Moreover, the knowledge of the signaling pathways elicited by the heterocomplex could be also relevant in understanding how to foster a CXCR4 signaling able to induce tissue regeneration.

## Conclusion

In conclusion, we have demonstrated that the CXCL12/HMGB1 heterocomplex engages the β-arrestin proteins differently from CXCL12, promoting a prompt availability of CXCR4 on the cell surface, allowing an enhanced response to the chemotactic cue.

## Data Availability Statement

The raw data supporting the conclusions of this article will be made available by the authors, without undue reservation.

## Author Contributions

GD’A and MA performed most of the experiments. GD’A, MA, ML, LP, DL, MB, CR, MT, AM, VC, and MU discussed project, experiments and results, and reviewed the manuscript. MR contributed to the analysis of the data, and reviewed the manuscript. GD’A, VC, and MU wrote the manuscript, and MU was responsible for general organization. All authors contributed to the article and approved the submitted version.

## Conflict of Interest

MB is founder and part owner of HMGBiotech, a company that provides goods and services related to HMGB proteins. The remaining authors declare that the research was conducted in the absence of any commercial or financial relationships that could be construed as a potential conflict of interest.

## Footnotes

1 http://bioinfo.ut.ee/primer3-0.4.0/
