# Supplementary material for: β-Arrestin1 and β-Arrestin2 Are Required to Support the Activity of the CXCL12/HMGB1 Heterocomplex on CXCR4
Source: Front Immunol. 2020 Sep 18;11:550824. doi: 10.3389/fimmu.2020.550824 (PMC7533569; doi:10.3389/fimmu.2020.550824)
Supplement: Supplementary file 1 [file Data_Sheet_1.docx]

Supplementary Material

β-arrestin1 and β-arrestin2 are required to support the activity of the CXCL12/HMGB1 heterocomplex on CXCR4

Gianluca D’Agostino, Marc Artinger, Massimo Locati, Laurent Perez, Daniel F. Legler, Marco E. Bianchi, Curzio Rüegg, Marcus Thelen, Adriano Marchese, Marco B.L. Rocchi, Valentina Cecchinato^*^ and Mariagrazia Uguccioni^*^

*** Correspondence:** Dr. Mariagrazia Uguccioni: [mariagrazia.uguccioni@irb.usi.ch](mailto:mariagrazia.uguccioni@irb.usi.ch); Dr. Valentina Cecchinato: [valentina.cecchinato@irb.usi.ch](mailto:valentina.cecchinato@irb.usi.ch)

Supplementary Material and Methods

**Flow cytometric analysis**

For surface staining of wt HeLa cells, cell suspensions were incubated for 30 min at 4ºC with the appropriate monoclonal antibody: CXCR4-APC (555976, BD Pharmingen, San Jose, CA ), or TLR-4-BrilliantViolet421 (312811, BioLegend, Dedham, MA). All primary antibodies were diluted 1:50. Samples were acquired by using BD LSR Fortessa flow cytometer from BD Biosciences (San Jose, CA) and analyzed with the FlowJo software (FLOWJO LLC, Ashland, OR).

**Supplementary Figures**


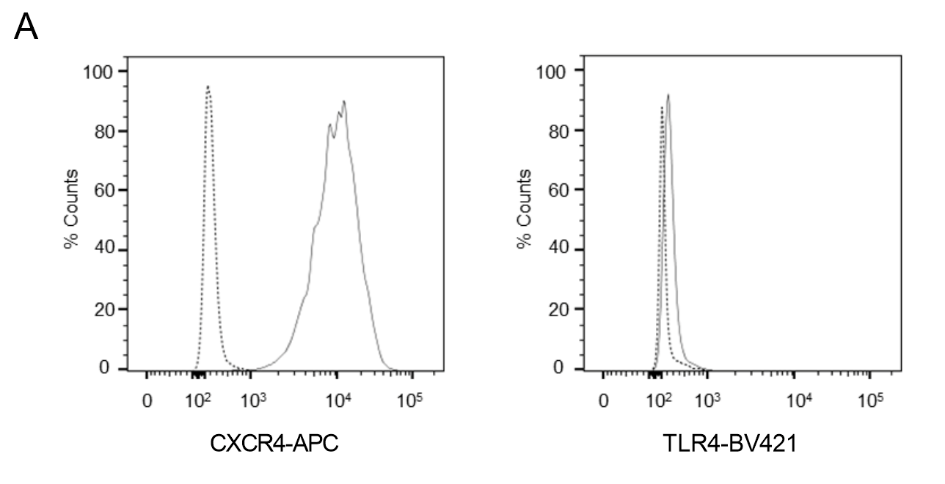


**Supplementary Figure S1.** Expression of CXCR4 and TLR-4 on wt HeLa cells. **(A)** Representative plot of CXCR4 and TLR-4 surface expression assessed by flow cytometry in HeLa cells. Dashed lines represent unstained negative controls.


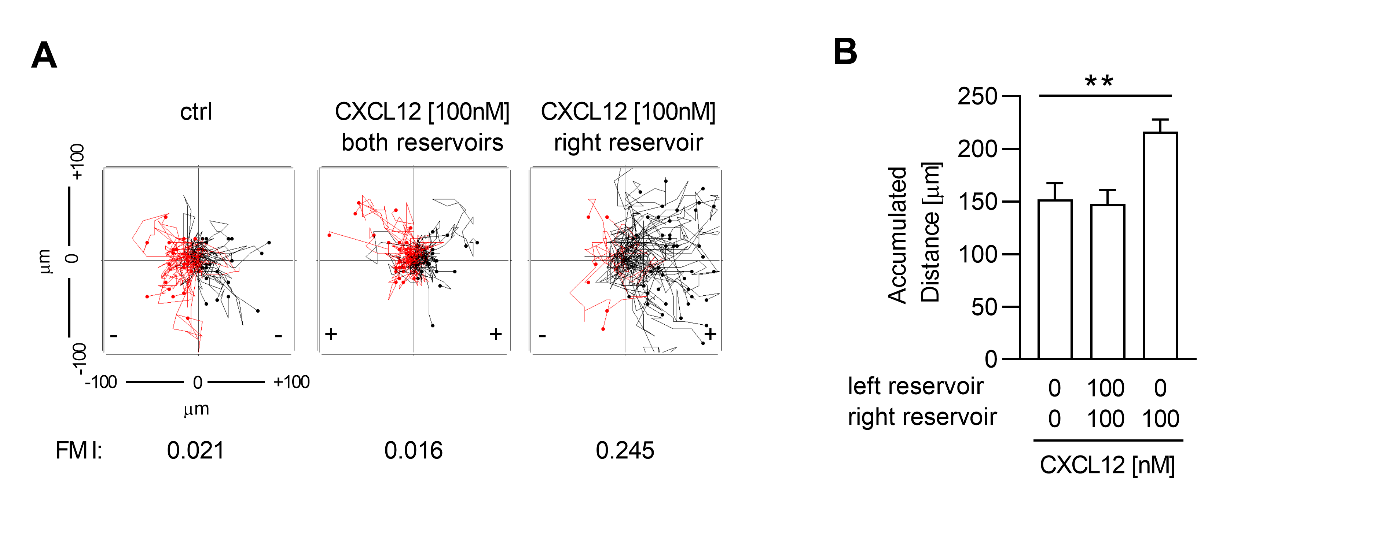


**Supplementary Figure S2.** Validation of HeLa cell migration assay in Ibidi µ-Slide Chemotaxis chambers. **(A)** HeLa cells were allowed to migrate in response to 100 nM CXCL12 for 18 hours. A negative control without any chemoattractant (-/-), and a control with the chemokine in both reservoirs of the chamber (+/+) were included. Representative spider plots, showing the trajectories of single tracked cells migrating to the right (black) or moving in the opposite direction (red), are shown. Black and red dots in the plots represent the final position of each single tracked cell. FMI: Forward Migration Index **(B)** Quantitative evaluation of the accumulated distance is shown. Mean ± SEM of 50 cells for each experimental condition. ** p < 0.001 using one-way ANOVA followed by Dunnett’s multiple comparison test.

**
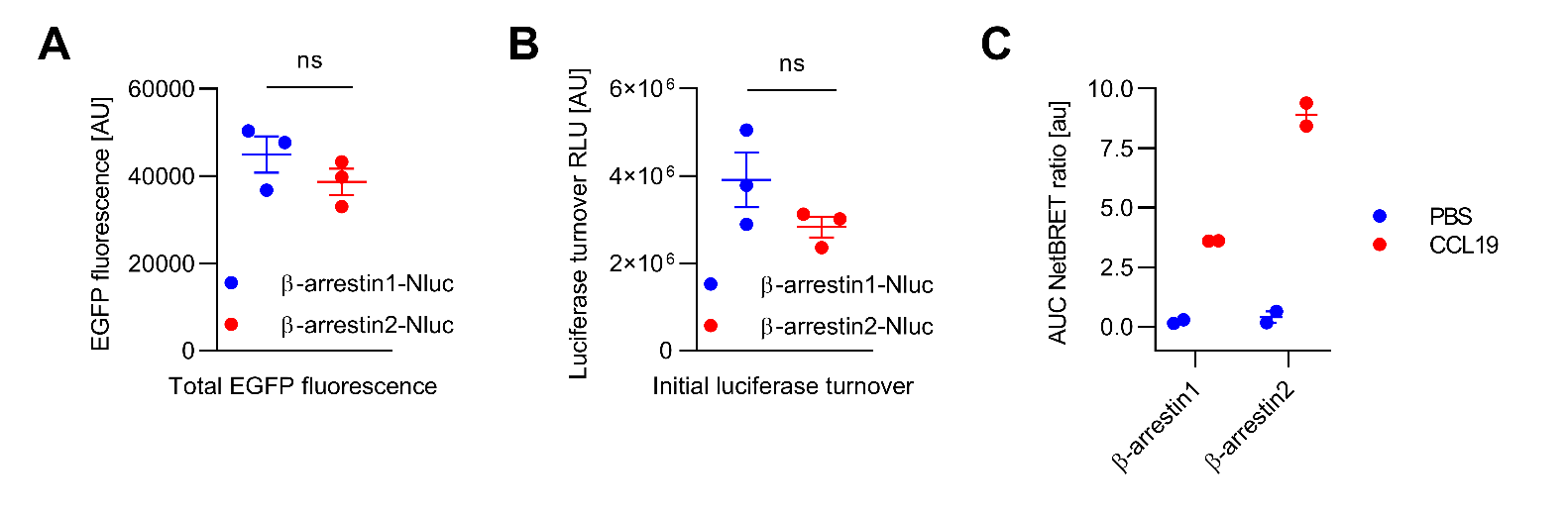
**

**Supplementary Figure S3.** Validation of BRET assay for the detection of β-arrestins recruitment to CXCR4. **(A)** Total fluorescence intensity of CXCR4-EGFP, and **(B)** luciferase turnover from the experiments shown in Figure 2A were comparable in HeLa cells co-expressing CXCR4-EGFP together with either β-arrestin1-nLuc (blue) or β-arrestin2-nLuc (red). Three independent experiments are shown. Mean ± SEM, ns (not significant) using unpaired, two tailed *t*-test. **(C)** Stimulating HeLa cells co-transfected with CCR7-EGFP instead of CXCR4-EGFP and either β-arrestin1-nLuc or β-arrestin2-nLuc revealed that both β-arrestin constructs are functional. Two independent experiments are shown (Mean ± SEM).


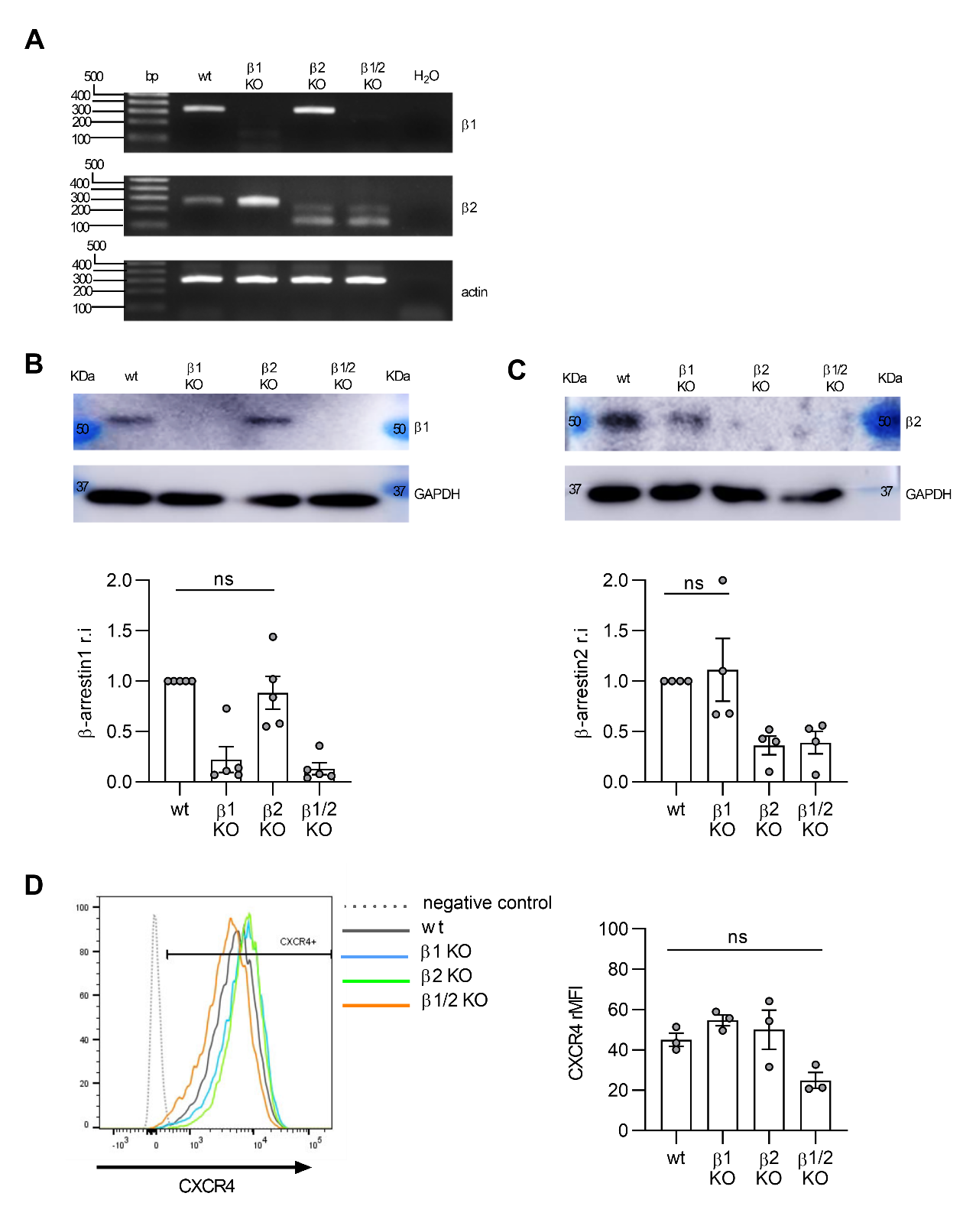


**Supplementary Figure S4.** Validation of β-arrestins KO generated by CRISPR/Cas9 in HeLa cells. **(A)** Representative PCR of *β-arrestin1* (β1), *β-arrestin2* (β2), and *actin* gene expression performed on cDNA from wild type and β-arrestins KO HeLa cells. **(B-C)** Representative Western blot analysis of β-arrestin1, β-arrestin2 and GAPDH protein expression in total cell extracts from wt and β-arrestins KO HeLa cells. Quantification of **(B)** β-arrestin1 (β1; n = 5), **(C)** β-arrestin2 (β2, n= 4) in all experiments performed. Mean ± SEM, ns (not significant) using unpaired, two tailed *t*-test. **(D)** Representative plot and quantification of CXCR4 surface expression assessed by flow cytometry in wt and β-arrestins KO HeLa cells. Dashed line represents the unstained negative control, mean ± SEM of 3 independent experiments, ns (not significant) using one-way ANOVA followed by Dunnett’s multiple comparison test.

Supplementary Tables

**Supplementary Table S1.** List of the primers used for the validation of the β‑arrestin KO clones by PCR. *β‑arrestin1* and *β-arrestin2* forward and reverse primers encompassing the RNA sequence region targeted by CRISPR/Cas9. The expression of the housekeeping gene *actin* was detected as internal control. The melting temperature specific for each primer is provided as well as the base pairs (bp) length of amplicons.

| cDNA | Forward Primer (5’-3’)  Melting Temperature (⁰C) | Reverse Primer (5’-3’)  Melting Temperature (⁰C) | Amplicon Length (bp) |
| --- | --- | --- | --- |
| *β-arrestin1* | TCACCGTCTACCTGGGAAAG  (60.5) | TTGGAGGGATCTCAAAGGTG  (58.4) | 312 |
| *β-arrestin2* | GCACCTATGGGGGAGAAAC  (59.5) | GTAGGTGGCGATGAACAGGT  (60.5) | 261 |
| *actin* | TCACCCACACTGTGCCCATCTACGA  (78) | CAGCGGAACCGCTCATTGCCAATGG (60) | 294 |
